# Improved recurrence rates and progression-free survival in primarily surgically treated oral squamous cell carcinoma – results from a German tertiary medical center

**DOI:** 10.1007/s00784-024-05644-z

**Published:** 2024-04-20

**Authors:** Ann-Kristin Struckmeier, Mayte Buchbender, Rainer Lutz, Marco Kesting

**Affiliations:** 1 Department of Oral and Cranio-Maxillofacial Surgery, Friedrich-Alexander-Universität Erlangen- Nürnberg (FAU), Glückstraße 11, 91054 Erlangen, Germany; 2 Comprehensive Cancer Center Erlangen-European Metropolitan Area of Nuremberg (CCC ER- EMN), Erlangen, Germany

**Keywords:** Oral squamous cell carcinoma, Survival, Recurrence, Free flap, Neck dissection

## Abstract

**Objectives:**

This study aimed to explore survival and recurrence patterns in patients undergoing primarily surgical treatment for oral squamous cell carcinoma (OSCC) at a high-volume tertiary medical center in Germany.

**Materials and methods:**

The study included 421 patients with primary OSCC who underwent radical tumor resection, neck dissection, and reconstruction with a free flap. Prognostic relevance of clinicopathological characteristics was assessed using Cox proportional-hazards models. Kaplan-Meier method estimated local recurrence-free survival, progression-free survival (PFS), and overall survival (OS), while the log-rank test compared survival outcomes between groups.

**Results:**

Recurrence manifested in 16.63% of the patients (70 patients), encompassing local recurrence in 54 patients (77.14%) and distant metastasis in 24 patients (34.28%). Neck recurrence occurred in only 1 patient (0.24%) on the contralateral side. The majority of recurrences occurred within the initial twelve months following primary tumor surgery (64.29%). Overall, the 5-year OS stood at 58.29%, while the 5-year PFS reached 72.53%. Patients with early recurrence within ≤ 12 months showed the least favorable prognosis (log-rank, all *p* < 0.001).

**Conclusions:**

Our findings show a significant decrease in recurrence rates and enhanced PFS at a high-volume tertiary medical center in Germany compared to previous studies. Local recurrence was the primary form observed, with most recurrences happening within the initial twelve months post-surgery. Opting for treatment at a high-volume center and devising therapy plans in interdisciplinary tumor boards may not only enhance OS but also contribute to improved PFS.

**Clinical relevance:**

These findings offer valuable insights for physicians regarding the post-treatment care of patients with OSCC. The results underscore the importance of frequent follow-up appointments, particularly during the initial year, and highlight the critical need for vigilance in monitoring for local recurrence.

**Supplementary Information:**

The online version contains supplementary material available at 10.1007/s00784-024-05644-z.

## Introduction

Oral squamous cell carcinoma (OSCC) constitutes about 90% of all malignant tumors in the oral cavity, with a worldwide incidence exceeding 350,000 cases [1, 2].

Traditional risk factors for the development of OSCC include smoking and excessive alcohol consumption [3]. Most cases of OSCC occur in males, with an average age of 65 years in the Western countries [4].

Usually, the primary approach for curative treatment of OSCC involves surgical intervention. In instances of advanced disease or high-risk pathological features, multimodal therapy, including adjuvant radiation or radiochemotherapy, should be contemplated [5, 6]. The surgical approach includes radical tumor resection, neck dissection, and reconstruction with a free flap. However, given its aggressive local invasion and propensity for metastasis, treating OSCC remains a formidable challenge within the realm of head and neck squamous cell carcinoma.

In spite of numerous advancements in diagnostic and therapeutic approaches over the last thirty years, the prognosis for patients with OSCC remains unfavorable, with documented 5-year overall survival (OS) hovering around 50 to 60% [7, 8]. The prognosis is significantly affected by recurrence, with rates of relapse ranging from 15 to 45% [9–11]. Hence, pinpointing the factors that influence the recurrence of OSCC has a pivotal role in clinical practice, particularly given that local and regional relapses contribute to approximately 90% of recurrences.

Notably, numerous studies have consistently indicated that receiving treatment at high-volume centers is associated with improved OS [12–15]. This correlation may stem from the critical importance of ensuring the adequacy of surgical resection for treatment success. High-volume centers typically demonstrate proficiency in performing reconstruction with free flaps following extensive resections to achieve negative margins. Furthermore, in current clinical practice, decisions concerning therapy are commonly deliberated within interdisciplinary tumor boards, which may be more readily available in high-volume centers. These tumor boards have demonstrated efficacy in advising treatment strategies for head and neck carcinomas, frequently leading to intensified therapy through the incorporation of multimodal treatments [16].

The study aimed to provide valuable insights into the current expected survival rates and recurrence patterns within a German high-volume tertiary medical center following a standardized treatment protocol for OSCC in line with the German guidelines.

## Materials and methods

### Study design and participants

The study included patients with primary OSCC who underwent surgical treatment, encompassing radical tumor resection and neck dissection, at a high-volume tertiary medical center in Germany between January 1, 2013, and May 31, 2023. The treatment protocol followed the current German guidelines, and all interventions were conducted based on recommendations established during tumor board meetings.

In our tertiary medical center, our primary surgical protocol for managing OSCC involves radical tumor resection, often complemented by reconstruction with a free flap when deemed necessary. Neck dissection is systematically performed in every patient following the established protocol: For patients without clinically evident neck metastases, we perform a ipsilateral supraomohyoid neck dissection that covers levels I to III, commonly referred to as selective neck dissection (SND). In instances where tumors are midline or approaching the midline, a bilateral SND is undertaken. In instances where there are preoperative, intraoperative (utilizing the frozen section technique), or postoperative indications of ipsilateral lymph node metastases, we conduct a modified radical neck dissection (MRND) on the ipsilateral side, accompanied by a contralateral selective neck dissection (SND). In cases of contralateral lymph node metastasis, a bilateral MRND is undertaken.

The decision for adjuvant therapy was based on the individual risk factors of each patient, adhering to the recommendations outlined in the German guidelines. Typically, patients with lymph node metastasis, perineural, vascular, or lymphatic invasion, scarce resection margins, or those with advanced tumor stages receive adjuvant radiotherapy after surgery. On the contrary, patients with positive resection margins or extranodal extension of lymph node metastases undergo adjuvant radiochemotherapy.

The follow-up schedule was organized as follows: In the initial year, clinical examinations were conducted every 6 weeks, transitioning to 3-month intervals in the second year. During the third and fourth years, follow-ups were scheduled at 6-month intervals, and in the fifth year, clinical examinations were performed annually. In addition, computed tomography scans were performed every 6 months during the first two years and then shifted to a 12-month interval in the subsequent three years.

The exclusion criteria encompassed patients with recurrent OSCC and squamous cell carcinoma of the lip. Patients who refused neck dissection or underwent a less extensive neck dissection than described above due to severe comorbidities were also excluded. Moreover, patients undergoing neoadjuvant treatment were excluded to ensure a homogeneous patient cohort.

The study design and methodlogy received approval from the Ethics Committee of the Friedrich-Alexander-University Erlangen-Nuremberg (Ethic votes: 23-185-Br, 23-186-Br). In compliance with national and institutional regulations, written informed consent was not deemed necessary.

The manuscript was prepared following the STROBE statement.

### Clinicopathological characteristics

Clinicopathological characteristics were extracted from the medical records. A systematic collection and evaluation were conducted for the following parameters: age, sex, tumor localization, TNM classification, Union for International Cancer Control (UICC) stages, depth of invasion, grading, resection margins, presence of perineural, vascular, and lymphatic invasion, and extranodal extension. Furthermore, we documented the time point of surgery, the latest follow-up, and, when available, the time point of recurrence and death.

The TNM classification underwent revision during the study period. To maintain consistency in our findings [17], we reclassified patients initially categorized under the 7^th^ TNM classification before 2017. As a result, all patients were categorized based on the 8^th^ TNM classification.

### Statistical analysis

Statistical analysis was conducted using the Statistical Package for the Social Sciences 28.0 (SPSS, Chicago, IL, USA).

Recurrence was defined as follows:

(1) Local recurrence – recurrence at the same anatomic site within 5 years after primary treatment; (2) Regional recurrence – lymph node metastases of the neck within 5 years after primary treatment; (3) Distant metastases – metastases elsewhere in the body, e.g., the lungs.

Correlation analysis utilized the Chi-square test.

To identify prognostic factors for survival, univariate Cox analysis was conducted, followed by a multivariate Cox analysis incorporating factors that exhibited significance in the univariate analysis.

Furthermore, local recurrence-free survival (LFS), progression-free survival (PFS), and OS were estimated using the Kaplan-Meier method. LFS was determined by calculating the duration from the day of surgery to the occurrence of local relapse. This duration was censored at the last recorded day when the patient remained alive without any evidence of recurrence. PFS was defined as the period from the day of surgery to the occurrence of local, regional or distant metastatic recurrence, and it was censored at the last recorded day when the patient was alive without any evidence of recurrence. OS as the duration from the day of resection to the event of death from any cause, and it was censored at the last day when the patient was still alive. We employed the log-rank test for comparing survival between groups.

Figures were also created using SPSS.

Generally, a p value < 0.05 was considered statistically significant.

## Results

### Clinicopathological characteristics

**Fig. 1:**
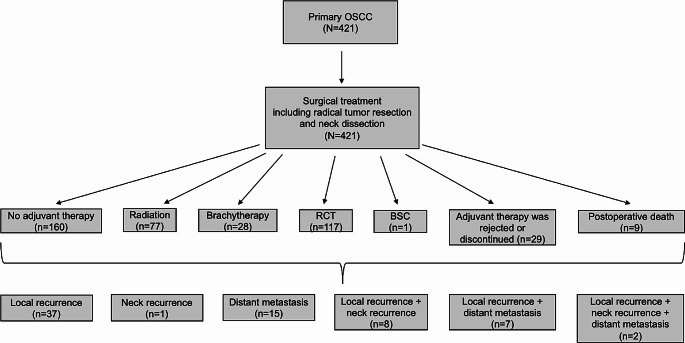
Flowchart of this study. *Abbreviations* RCT: radiochemotherapy, BSC: best supportive care

Our final study cohort consisted of 421 patients diagnosed with primary OSCC, all of whom underwent radical tumor resection, neck dissection, and reconstruction with a free flap. 60.81% (256 out of 421) of the patients received adjuvant treatment, such as brachytherapy, radiation, or radiochemotherapy. However, 29 patients (6.89%) either opted to forgo adjuvant therapy or did not complete it, despite its recommendation. Figure 1 illustrates the flowchart of this study.

Most patients in the cohort were male (260 patients, 61.76%). The median age of the patient cohort ranged from 31 to 93 years, with a median age of 64 years. The primary tumor localizations were the floor of the mouth (150 patients, 35.63%) and the tongue (105 patients, 24.94%).

The distribution of pathological tumor stages was as follows: 153 (36.34%) in T1, 108 (25.65%) in T2, 66 (15.68%) in T3, and 94 (22.44%) in T4a.

Histopathological examination revealed the absence of lymph node metastasis in 278 patients (66.03%), while 43.97% presented with metastatic disease.

Histopathological analysis unveiled that half of the patients had moderately differentiated carcinomas (48.45%, 204 patients), while 31.35% exhibited poorly differentiated carcinomas (132 patients), and only 8.80% displayed well-differentiated carcinomas (37 patients). Furthermore, histopathological analysis revealed lymphatic invasion in 5.70% (24 patients), vascular invasion in 1.90% (8 patients), and perineural invasion in 15.68% of the tumors (66 patients). Microscopically positive margins were observed in 1.42% of cases (6 patients).

### Correlation analysis

Correlation analysis was conducted to discern relationships between clinicopathological characteristics and the likelihood of recurrence. The analysis revealed a significant correlation between recurrence and the pathological tumor stage, nodal stage, UICC stage, grading, lymphatic invasion (Chi-square, all *p* < 0.001), and perineural invasion (Chi-square, *p* = 0.026). Additional information regarding the correlation analysis is available in Table S1.

### Patterns of recurrence

**Fig. 2:**
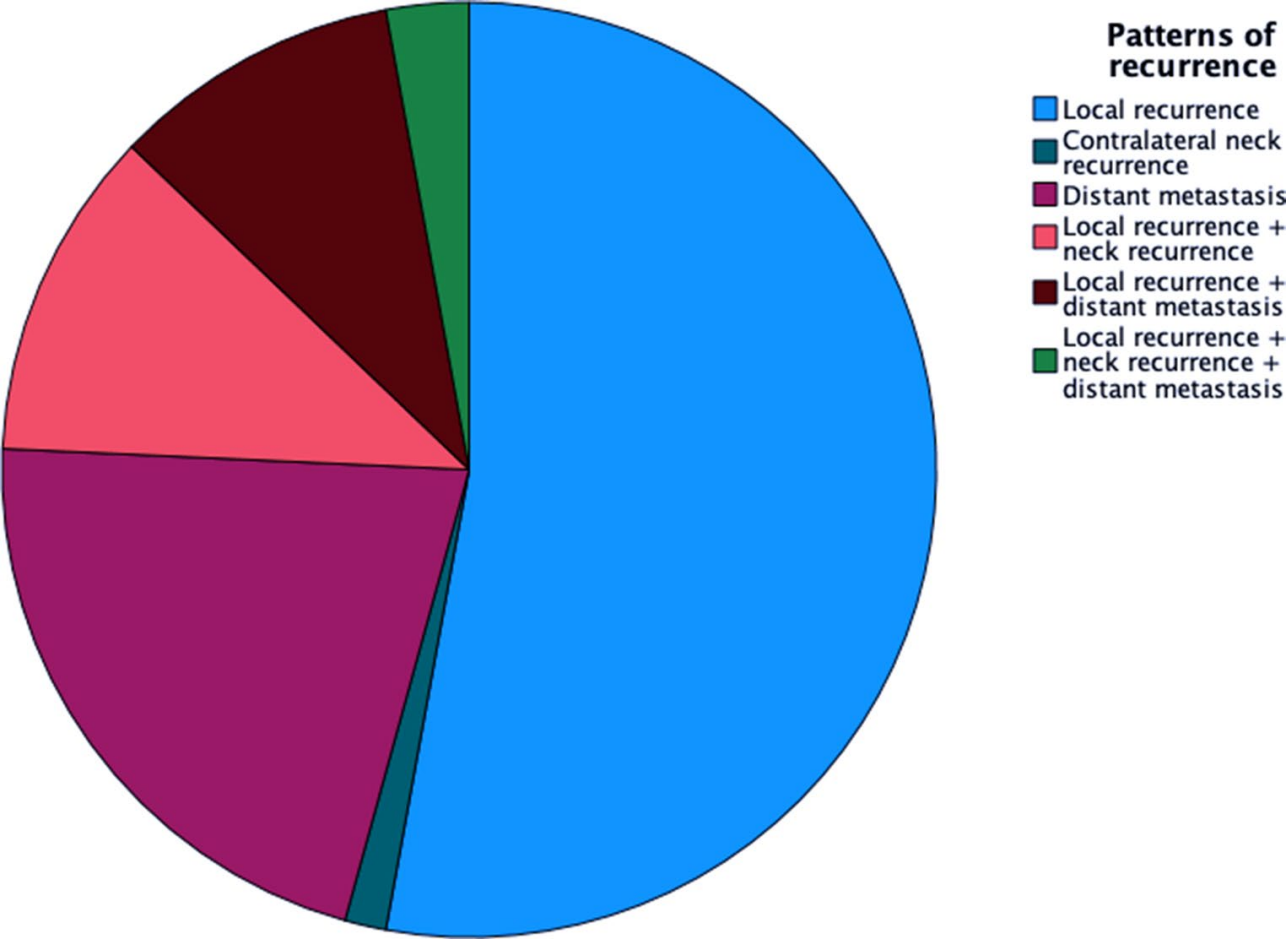
Patterns of recurrence after surgically treated primary oral squamous cell carcinoma

The overall recurrence rate was 16.63% (70 patients), encompassing local recurrence in 37 patients (8.79%), contralateral neck recurrence in 1 patient (0.24%), and distant metastasis in 15 patients (3.56%). Concomitant local recurrence and neck recurrence were observed in 8 patients (1.90%), while local recurrence combined with distant metastasis occurred in 7 patients (1.66%). Furthermore, 2 patients (0.48%) experienced the simultaneous presence of local recurrence, neck recurrence, and distant metastasis. A comprehensive breakdown of recurrence rates is provided in Table S2 and Fig. 2.

### Recurrence interval

Among the 70 patients who experienced recurrence (local, regional, and/or distant metastasis), 20 patients (28.57%) had a recurrence within the first 6 months, with an additional 25 patients (35.71%) encountering recurrence during the period from the 6^th^ to the 12^th^ month. In total, 45 patients (64.29%) faced a recurrence within the initial year following surgical therapy. Moreover, an additional 10 patients (14.28%) had a recurrence between the 12^th^ and 18^th^ months, with recurrences becoming infrequent in subsequent stages. Overall, 82.86% of recurrences manifested within the initial two years.

The mean time interval from surgical treatment to recurrence was 16.43 ± 19.76 months.

**Fig. 3:**
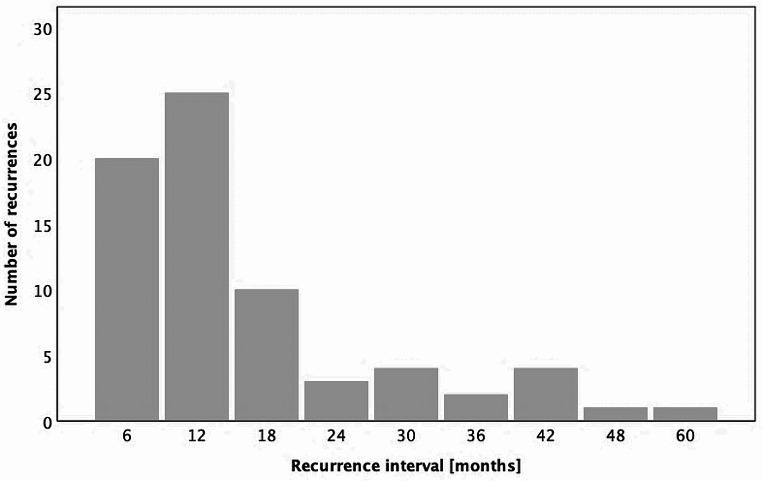
Distribution of recurrence interval after surgically treated primary oral squamous cell carcinoma

The distribution of the recurrence interval is depicted in Fig. 3.

### Impact of time point of recurrence on overall survival

**Fig. 4:**
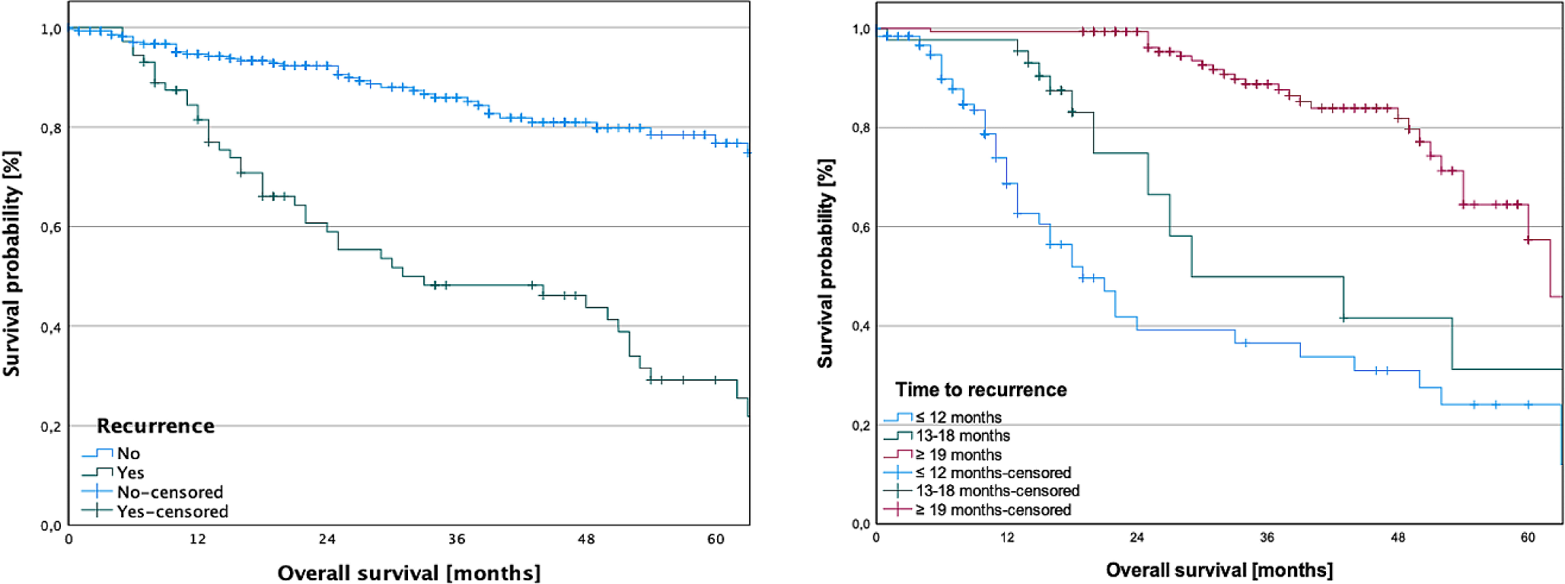
Impact of recurrence and time to recurrence on survival in surgically treated primary oral squamous cell carcinoma. Survival was significantly worse when relapse occurred (log-rank, *p* < 0.001). Furthermore, there were notable variations in survival outcomes among patients experiencing relapse within different intervals: ≤ 12 months, 13–18 months, and ≥ 19 months, with the least favorable prognosis observed in patients with early recurrence within ≤ 12 months (log-rank, all *p* < 0.001)

Subsequently, we investigated how the timing of relapse influenced OS. Generally, survival was significantly worse when recurrence occurred (log-rank, *p* < 0.001). Furthermore, there were notable variations in survival outcomes among patients experiencing relapse within different intervals: ≤ 12 months, 13–18 months, and ≥ 19 months, with the least favorable prognosis observed in patients with early recurrence within ≤ 12 months (log-rank, all *p* < 0.001). Kaplan-Meier curves illustrating the variation in survival based on the time point of recurrence are presented in Fig. 4.

### Local recurrence-free survival

**Fig. 5:**
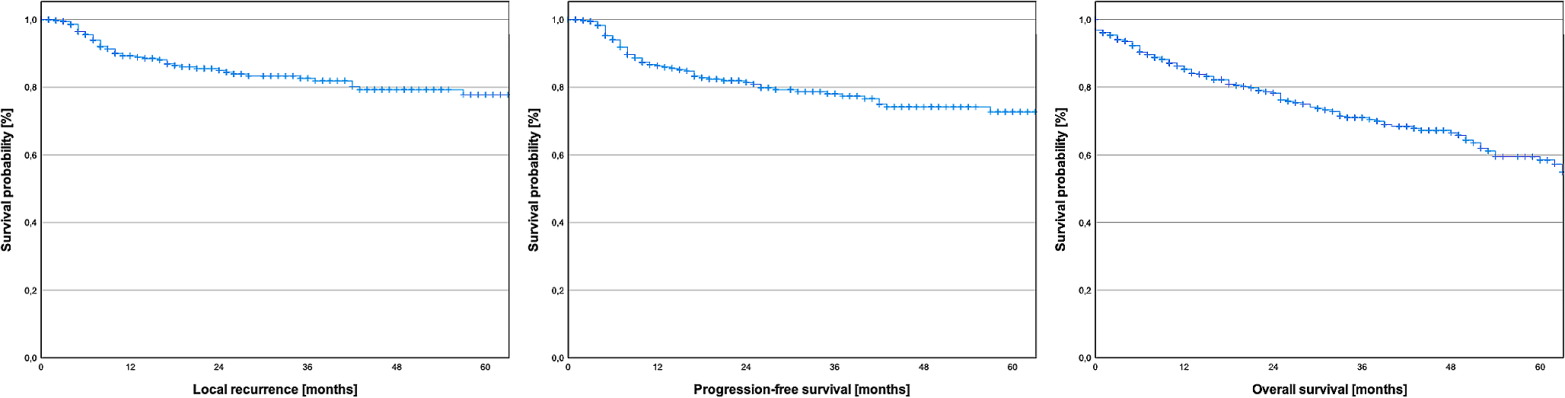
Survival rates after surgically treated primary oral squamous cell carcinoma. The 5-year-local recurrence-free survival, progression-free survival, and overall survival were 77.74%, 72.53%, and 58.29%, respectively

**Fig. 6:**
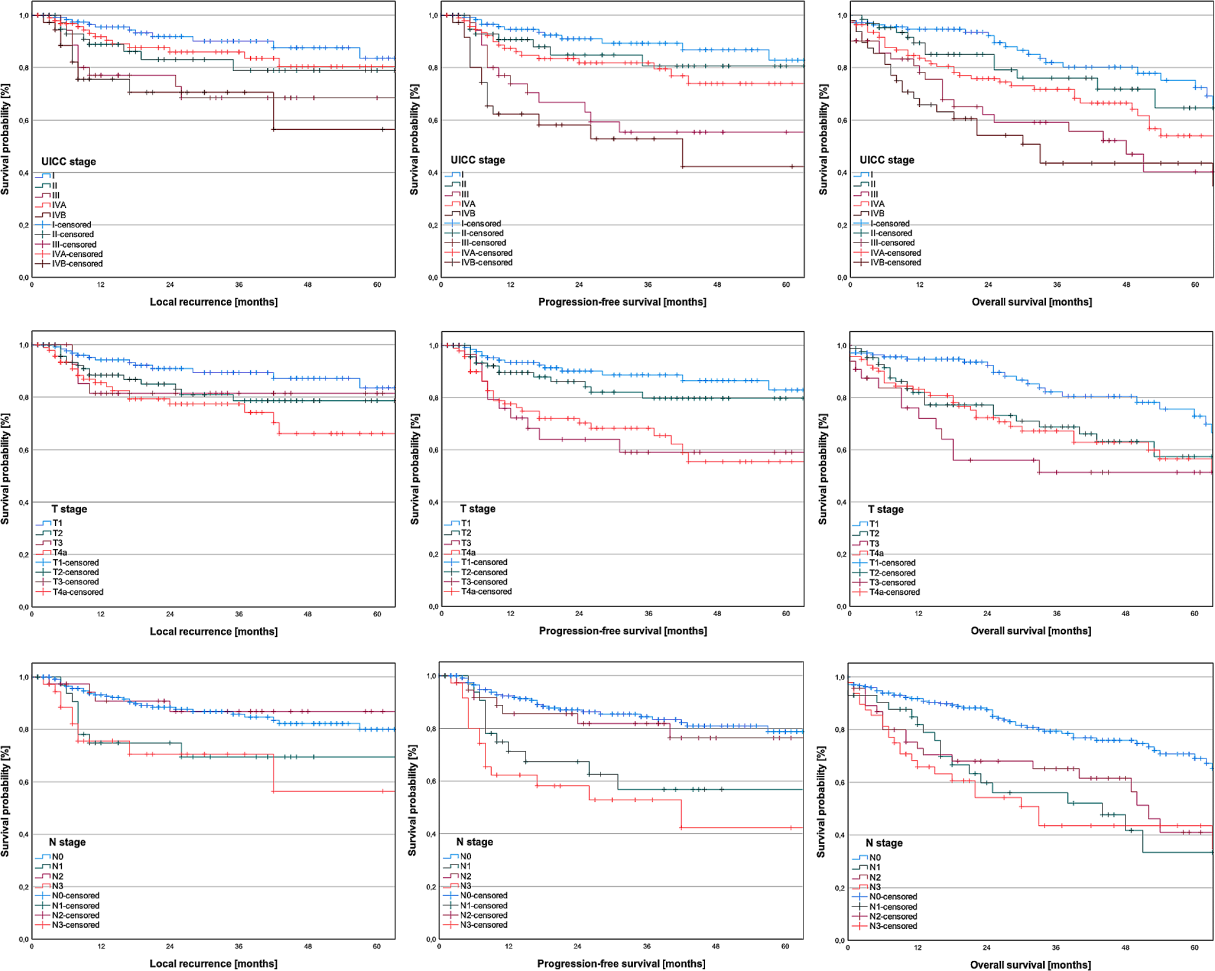
Survival rates based on UICC stages, tumor stages, and nodal stages according to the 8th TNM edition in primarily surgically treated oral squamous cell carcinoma. The 5-year local recurrence-free survival for UICC stages I-IVB was 85.24%, 80.22%, 68.17%, 81.47%, and 57.01%, respectively. Similarly, the 5-year progression-free survival stratified by UICC stages was observed to be 83.84%, 80.97%, 55.37%, 73.73%, and 40.13%, respectively. On the contrary, the 5-year overall survival was observed to be 74.46%, 64.20%, 39.23%, 52.22%, and 42.25%, respectively

Subsequently, our aim was to assess the 5-year LFS in our patient cohort, which was found to be 77.74%. The Kaplan-Meier curve for LFS is displayed in Fig. 5. To provide a more detailed breakdown, the 5-year LFS for UICC stages I-IVB was 85.24%, 80.22%, 68.17%, 81.47%, and 57.01%, respectively. Figure 6 illustrates Kaplan-Meier curves depicting LFS based on tumor stages, nodal stages, and UICC stages.

In the subsequent step to identify prognostic factors for LFS, we conducted univariate Cox analysis, followed by multivariate Cox analysis, incorporating factors that demonstrated significance in the univariate analysis.

In univariate Cox regression analysis, the nodal stage (*p* = 0.009), tumor stage (*p* = 0.005), grading (*p* = 0.002), lymphatic invasion (*p* = 0.002), and resection margins (*p* = 0.012) were identified as prognostic factors for LFS. Subsequent multivariate Cox regression analysis confirmed grading (*p* = 0.041) and resection margins (*p* = 0.017) as independent prognostic factors. The outcomes of both univariate and multivariate analyses are presented in Table S3.

### Progression-free survival

Next, we aimed to determine 5-year PFS in our patient cohort. The 5-year PFS was determined to be 72.53% and the Kaplan-Meier curve for PFS can be found in Fig. 5. When stratified by UICC stages I-IVB, the 5-year PFS was observed to be 83.84%, 80.97%, 55.37%, 73.73%, and 40.13%, respectively. Figure 6 presents Kaplan-Meier curves illustrating PFS according to tumor stages, nodal stages, and UICC stages.

Following that, we examined factors that significantly impact PFS. Univariate Cox regression analysis demonstrated significant prognostic factors including nodal stage, tumor stage, grading, lymphatic invasion (all *p* < 0.001), perineural invasion (*p* = 0.044), and vascular invasion (*p* = 0.009). Subsequent multivariate Cox regression analysis confirmed tumor stage (*p* = 0.008), grading (*p* = 0.016), and lymphatic invasion (*p* = 0.015) as independent prognostic factors. The outcomes of univariate and multivariate analyses are depicted in Table S4.

### Overall survival

In the final step, our objective was to determine 5-year OS and to identify significant factors for OS. The 5-year OS was determined to be 58.29%. The corresponding Kaplan-Meier curve for OS can be found in Fig. 5. When stratified by UICC stages I-IVB, the 5-year OS was observed to be 74.46%, 64.20%, 39.23%, 52.22%, and 42.25%, respectively. Kaplan-Meier curves depicting OS based on tumor stages, nodal stages, and UICC stages are shown in Fig. 6.

Univariate Cox regression analysis identified several prognostic factors, including age, nodal stage, tumor stage, lymphatic invasion (all *p* < 0.001), grading (*p* = 0.006), perineural invasion (*p* = 0.020), venous invasion (*p* = 0.020), and resection margins (*p* = 0.018). However, multivariate Cox regression only confirmed age (*p* < 0.001) as an independent prognostic factor. Please refer to Table S5 for a more comprehensive breakdown of information regarding univariate and multivariate analyses.

## Discussion

In this retrospective study, we investigated survival outcomes and recurrence patterns in a cohort of 421 patients diagnosed with primary OSCC. Our findings revealed a noteworthy reduction in recurrence rates compared to previous studies, accompanied by an improvement in PFS.

We demonstrated a recurrence rate of 16.63% in our study. However, following curative treatment of OSCC, overall recurrence rates are typically higher, ranging from 21 to 52% [18–22]. For instance, Jerjes et al. documented a recurrence rate of 37.4% in a cohort of 115 patients with small T1/T2 tumors [23]. Nonetheless, a noteworthy challenge in comparing previous studies stems from variations in treatment regimens, e.g., decisions regarding adjuvant therapy. However, Carvalho et al. conducted the largest primary research study to date, encompassing data from 2067 patients between 1954 and 1998. They reported an overall recurrence rate of 52.2% [19]. On the contrary, Brown et al. reported a local and regional recurrence rate of 21%, encompassing patients who underwent either exclusive surgery or a combination of surgery and adjuvant radiotherapy [20]. Liu et al. reported a local and regional recurrence rate of 25% in patients treated with surgery alone [24]. In our cohort, patients received primary surgical treatment including radical tumor resection, neck dissection, and reconstruction with free flap. None of the patients received neoadjuvant therapy. The decision for adjuvant therapy was based on the individual risk factors of each patient, adhering to the recommendations outlined in the German guidelines.

In the subsequent phase, we scrutinized the patterns of recurrence, as understanding these is crucial for the early detection of recurrent disease, assessing resectability, and preoperative planning. When classified by the type of recurrence, previously reported rates of local, regional, and locoregional recurrences in OSCC typically range from 30.2 to 61.6%, 24–51.1%, and 4.1–16.3%, respectively [12–18]. However, in our study cohort, local recurrences were predominant, accounting for 8.79% of cases. Regional recurrence was observed in one patient who, surprisingly, initially presented with a unilateral pT1 pN0 tumor localized at the tongue. This patient received unilateral SND and subsequently developed contralateral lymph node metastases one year after primary treatment. Locoregional recurrence was observed in only 8 patients, constituting 1.90% of the entire patient cohort. The minimal occurrence of local recurrence can be attributed to the inclusion criteria in our study, which focused exclusively on patients undergoing radical tumor resection with concomitant reconstruction with a free flap. The incorporation of microvascular reconstruction facilitated more extensive resections, ensuring negative margins. This perspective was corroborated by Hsieh et al., who conducted a comparative analysis among patients diagnosed with advanced stage IV OSCC who underwent ablative tumor resection, with or without free flap reconstruction. Their group noted a higher occurrence of advanced tumors in the group undergoing free flap reconstruction, whereas the group without free flap reconstruction demonstrated a higher incidence of positive margins (17.2% vs. 23.5%). Despite the advanced cancer stages observed in patients necessitating free flap reconstruction, their survival rates and cancer recurrence outcomes were comparable to those of patients who did not undergo this reconstructive procedure [25].

We examined the impact of recurrence on OS. In general, survival outcomes were significantly worse when recurrence occurred (log-rank, *p* < 0.001). This finding aligns with Camisasca et al., who emphasized a notable difference in the 5-year OS between patients with recurrent OSCC (30%) and those without (92%, *p* < 0.001) [4]. However, Mücke et al. reported that patients with local recurrence had a better prognosis compared to those with regional recurrence (5-year OS: 37.5% vs. 21.5%, respectively) [26]. Notably, our cohort was primarily characterized by local recurrence, with regional recurrence observed in only one case. The low incidence of regional and locoregional recurrence can probably be ascribed to the systematic implementation of elective or therapeutic neck dissection in every patient of our study, as elucidated in the methods section. Previous research has indicated that OSCC patients who undergo elective neck dissection experience improved disease-free survival (DFS) and OS compared to those who undergo surveillance and subsequent therapeutic neck dissection [26–28]. For example, D’Cruz et al. described that after 3 years, elective neck dissection resulted in an enhanced OS (80.0%) compared to therapeutic dissection (67.5%), with a hazard ratio for death of 0.64 in the elective-surgery group. Additionally, patients in the elective-surgery group exhibited a higher rate of DFS compared to those in the therapeutic-surgery group (69.5% vs. 45.9%, *P* < 0.001) at that time [27]. Furthermore, Ren et al. conducted a meta-analysis comparing the effects of END and therapeutic neck dissection on survival and recurrence. Their analysis of five randomized controlled trials demonstrated that DFS was significantly higher in the END group than in the therapeutic neck dissection group (Risk Ratio: 1.33). Furthermore, their meta-analysis of four RCTs revealed a higher OS in the END group compared to the therapeutic neck dissection group, with a significant inter-group difference (Risk Ratio: 1.18). They concluded that performing elective neck dissection at the time of primary tumor resection provides both DFS and OS benefits in patients with clinically node-negative oral cancer [29].

Up to 76% of recurrences occur within the initial two years [30]. Some studies even suggest recurrence rates of up to 86% within the first year [31]. These early recurrences have been linked with a less favorable prognosis compared to late relapses [32, 33]. Hence, we examined the timing of recurrence in our patient cohort and its impact on survival.

In our study, 64.29% of all recurrences manifested after the first 12 months, with an additional 14.28% occurring between the 12^th^ and 18^th^ months. The frequency of recurrences decreased in subsequent stages, and, overall, 82.86% of recurrences occurred within the initial two years.

The mean time interval from surgical treatment to recurrence was 16.43 ± 19.76 months, consistent with the diverse time intervals documented in existing literature, ranging from 1 month to 60 months [31, 34].

As mentioned earlier, survival significantly deteriorated in our patient cohort when recurrence occurred (log-rank, *p* < 0.001). This finding aligns with earlier results [32, 35]. Nevertheless, there exists variability in determining the optimal cutoff value for distinguishing early recurrence, associated with a poor prognosis, from late recurrence, which is linked to a more favorable prognosis. While some studies identify 18 months as the optimal cutoff value and report significantly lower OS for recurrences occurring < 18 months compared to those occurring > 18 months (20.5% vs. 42.3% and 27.6% vs. 38.2%, respectively) [32, 36], others, such as Liao et al., have determined an optimal cutoff value of 10 months [35]. Yet, within our study group, substantial differences in survival outcomes emerged among patients encountering recurrence within distinct intervals: ≤ 12 months, 13–18 months, and ≥ 19 months. The most unfavorable prognosis was evident in patients experiencing early recurrence within ≤ 12 months (log-rank, all *p* < 0.001).

Hence, the prognosis is bleaker in cases of early recurrence, necessitating careful consideration in post-treatment surveillance. Our findings reinforce the existing follow-up protocol, emphasizing the necessity for frequent clinical examinations and computed tomographies during the first 2 years post-treatment. Afterward, the frequency gradually diminishes until the completion of the 5^th^ year.

The subsequent objective was to pinpoint factors associated ^wi^th recurrence. Our correlation analysis revealed a significant association between relapse in OSCC patients and pathological tumor stage, nodal stage, UICC stage, grading, lymphatic invasion (Chi-square, all *p* < 0.001), and perineural invasion (Chi-square, *p* = 0.026). These findings align with previous research. However, some studies reported a significant correlation with vascular invasion and close or positive resection margins [4, 9, 23, 37, 38]. Nevertheless, these histological characteristics showed a trend toward significance in our analysis (Chi-Square, *p* = 0.054 and *p* = 0.096, respectively). The low number of microscopically positive margins in our patient cohort might contribute to this result.

Next, we examined the 5-year PFS in our patient cohort. The 5-year PFS was determined to be 72.53%. When stratified by UICC stages I-IVB, the 5-year PFS was observed to be 83.84%, 80.97%, 55.37%, 73.73%, and 40.13%, respectively. The PFS in our cohort was relatively low when compared to others. For example, Manuel et al. reported a 5-year PFS of 57.4% [39]. This improvement in comparison to other studies might be attributed to the fact, as mentioned in the introduction, that therapy took place at a high-volume center with experienced surgeons and therapy was planned as decided in interdisciplinary tumor boards. The findings presented by Liu et al. underscore the importance of centralizing management in high-volume centers under the care of experienced surgeons to enhance patient survival rates. In their analysis, surgeon volume emerged as the most influential factor in improving patient outcomes. Notably, in their study, patients treated by high-volume surgeons experienced a significant reduction in mortality rates, with approximately a 60% decrease compared to those treated by low-volume surgeons [40].

Subsequently, we conducted an analysis to explore the impact of tumor-specific and patient-related risk factors on PFS in OSCC patients. Our investigation revealed tumor stage (*p* = 0.008), grading (*p* = 0.016), and lymphatic invasion (*p* = 0.015) as independent prognostic factors for PFS. In contrast, other studies have highlighted nodal stage, tumor stage, and resection margins as the most common prognostic factors for tumor recurrence [41, 42]. However, the results regarding the impact of nodal stage might be attributed to the limited observation of neck recurrence in our cohort.

As previously mentioned for correlation analysis, positive resection margins are a well-established risk factor for disease recurrence and are described to compromise the 5-year DFS in patients with head and neck squamous cell carcinoma [43]. Moreover, the status of resection margins plays a crucial role in determining the need for adjuvant therapy in patients [6]. However, as already stated, the occurrence of microscopically positive margins was very low in our patient cohort, potentially influencing this observation. Nevertheless, we identified resection margins as independent prognostic factor regarding LFS (*p* = 0.017).

The correlation between histopathological grading and PFS, as well as recurrence, remains a subject of debate with controversial results in the literature. Safi et al. confirmed grading as a risk factor for locoregional recurrence in OSCC [44]. On the contrary, Dik et al. found grading to have little predictive value in early-stage OSCC [45]. However, Xu et al. identified pathological grade as an independent risk factor for early-stage OSCC but not for advanced stages [46]. Overall, the importance of grading regarding PFS and recurrence remains contentious.

Similarly, the influence of age on recurrence and, consequently, PFS remains a subject of controversy in the literature. In our study, we did not identify a substantial impact of age on these outcomes. However, Friedlander et al. reported a higher rate of locoregional recurrence among patients younger than 40 years with OSCC localized at the tongue compared to older patients [14]. Conversely, Davison et al. concluded that increasing age predicted worse DFS [15].

5-year-OS of OSCC hovers around 50 to 60%, with a decline noted in advanced UICC stages [47]. Our study assessed the 5-year OS of OSCC patients following a standardized treatment protocol, revealing a rate of 62.5%. The survival rate aligns with previous findings, such as the 62% overall survival (OS) reported by Ansarin et al. [40], and surpasses the 48% reported by Sklenicka et al. [38]. However, the survival rate in our patient cohort may be influenced by the relatively advanced age of our patients and the patients presenting with multiple comorbidities commonly encountered in a tertiary medical center.

However, we conducted an analysis to explore the impact of tumor-specific and patient-related risk factors on OS in OSCC patients. Several factors, including age, tumor stage, nodal stage, lymphatic invasion (all *p* < 0.001), histopathological grading (*p* = 0.016), perineural invasion (*p* = 0.020), vascular invasion (*p* = 0.020), and resection margins (*p* = 0.018), were identified as significant factors. However, only age was confirmed as an independent prognostic factor in multivariate analysis (*p* < 0.001). Akin to many other cancers, survival tends to be higher among younger patients when compared to the older ones [48].

Nevertheless, the primary prognostic factor in OSCC is the presence of cervical lymph node metastasis, which leads to a 50% reduction in OS [3, 4]. Additionally, tumor stage and nodal stage, integral components of the TNM classification, serve as reliable basis for clinicians to assess patient prognosis and guide therapeutic decision-making. In our analysis, we identified tumor and nodal stages as prognostic factors in univariate analysis; however, their status as independent prognostic factors were not confirmed in multivariate analysis. Nevertheless, these results could be ascribed to the highly significant impact of age within our cohort.

As for PFS, there is a debate regarding the importance of histopathological grading. While grading has been reported by several authors as a significant prognostic factor for PFS and OS [49, 50], others have found no prognostic value for clinical outcome and response to treatment [51, 52]. A pivotal point in this discussion is the potentially subjective nature of histopathological grading, leading to notable inter- and intraobserver variabilities. Particularly, discrepancies in differentiation within various regions of the tumor, especially at the tumor margin and central aspects, add to the difficulty of consistently determining grading [53]. In summary, grading exhibits promise for informing a risk-stratified follow-up plan and warrants consideration in future prospective trials.

### Limitations of this study

The main limitations of our study involve the sample size and retrospective methodology. Previous investigations regarding survival in OSCC patients often faced challenges related to smaller sample sizes or heterogeneous data. As previously mentioned, our study specifically focused on patients who underwent primary surgical therapy for primary OSCC, and all participants underwent concomitant neck dissection. Additionally, we excluded patients undergoing neoadjuvant therapy, resulting in a highly homogeneous patient cohort. Furthermore, in contrast to earlier studies, we employed the 8^th^ TNM classification, published in 2017, to stage all patients, ensuring a uniformly classified patient cohort. However, the retrospective nature of our study Implies that the accuracy of data acquisition heavily depends on the precision of clinical records.

## Conclusion

Our results revealed a noteworthy reduction in recurrence rates and an improvement in PFS within a high-volume tertiary medical center in Germany, as compared to findings from previous studies. Local recurrence emerged as the predominant form of recurrence. Importantly, the majority of recurrences occurred within the initial twelve months following primary tumor surgery, emphasizing the necessity for closely spaced follow-up intervals during this critical period. The observed improvements may, in part, be attributed to the approach employed, where all patients underwent either therapeutic or elective neck dissection. Moreover, opting for treatment at a high-volume center and deliberating treatment decisions in interdisciplinary tumor boards may not only enhance OS but also contribute to improved PFS.

### Electronic supplementary material

Below is the link to the electronic supplementary material.

Supplementary Material 1

## Data Availability

The data that support the findings of this study are available from the corresponding author upon reasonable request.

## Abbreviations

CI: confidence interval
DFS: disease-free survival
END: elective neck dissection
LFS: local recurrence-free survival
MRND: modified radical neck dissection
OS: overall survival
OSCC: oral squamous cell carcinoma
PFS: progression-free survival
SND: selective neck dissection
TNM: tumor, node, metastasis
UICC: Union for International Cancer Control
