# Supplementary material for: Improved recurrence rates and progression-free survival in primarily surgically treated oral squamous cell carcinoma – results from a German tertiary medical center
Source: Clin Oral Investig. 2024 Apr 20;28(5):262. doi: 10.1007/s00784-024-05644-z (PMC11032275; doi:10.1007/s00784-024-05644-z)
Supplement: Supplementary file 1 — Supplementary Material 1 [file 784_2024_5644_MOESM1_ESM.docx]

**Supplemental material:** **Improved recurrence rates and progression-free survival in primarily surgically treated oral squamous cell carcinoma – results from a German tertiary medical center**

Ann-Kristin Struckmeier MD, DMD, Mayte Buchbender DMD, Rainer Lutz MD, DMD, Marco Kesting MD, DMD

| **Characteristics** | | **Disease-free** | **Recurrence** | **Correlation (Chi-square)** |
| --- | --- | --- | --- | --- |
| **Sex** | **Male** | 184 (80.70) | 44 (19.30) | 0.786 |
|  | **Female** | 107 (79.26) | 28 (20.74) |  |
| **Age** | **< 65 years** | 169 (80.84) | 41 (19.52) | 0.894 |
|  | **≥ 65 years** | 122 (79.74) | 31 (20.26) |  |
| **Tumor stage** | **T1** | 126 (90.00) | 14 (10.00) | <0.001* |
|  | **T2** | 79 (83.16) | 16 (16.84) |  |
|  | **T3** | 22 (64.71) | 12 (35.29) |  |
|  | **T4a** | 64 (68.09) | 30 (31.91) |  |
| **Nodal stage** | **N0** | 219 (86.56) | 34 (13.44) | <0.001* |
|  | **N1** | 25 (67.57) | 12 (32.43) |  |
|  | **N2a** | 5 (62.50) | 3 (37.50) |  |
|  | **N2b** | 17 (80.95) | 4 (19.05) |  |
|  | **N2c** | 6 (75.00) | 2 (25.00) |  |
|  | **N3b** | 19 (52.78) | 17 (47.22) |  |
| **UICC stage** | **I** | 117 (90.70) | 12 (9.30) | <0.001* |
|  | **II** | 51 (85.00) | 9 (15.00) |  |
|  | **III** | 27 (65.85) | 14 (34.15) |  |
|  | **IVA** | 77 (79.38) | 20 (20.62) |  |
|  | **IVB** | 19 (52.78) | 17 (47.22) |  |
| **Lymphovascular invasion** | **L0** | 277 (82.20) | 60 (17.80) | <0.001* |
|  | **L1** | 12 (50.00) | 12 (50.00) |  |
| **Vascular invasion** | **V0** | 285 (80.74) | 68 (19.26) | 0.054 |
|  | **V1** | 4 (50.00) | 4 (50.00) |  |
| **Perineural invasion** | **Pn0** | 243 (82.37) | 52 (17.63) | 0.026* |
|  | **Pn1** | 46 (69.70) | 20 (30.30) |  |
| **Grading** | **G1** | 35 (94.59) | 2 (5.41) | <0.001* |
|  | **G2** | 162 (86.17) | 26 (13.83) |  |
|  | **G3** | 90 (68.18) | 42 (32.82) |  |
| **Resection margins** | **R0** | 286 (80.56) | 69 (19.44) | 0.096 |
|  | **R1** | 3 (50.00) | 3 (50.00) |  |

**Table S1.** Prevalence of recurrence according to clinicopathological characteristics

| **Local recurrence** | 37 (8.79%) |
| --- | --- |
| **Regional recurrence** | 1 (0.24%) |
| **Distant metastasis** | 15 (3.56%) |
| **Locoregional recurrence** | 8 (1.90%) |
| **Local recurrence and distant metastasis** | 7 (1.66%) |
| **Locoregional recurrence + distant metastasis** | 2 (0.48%) |
| **Overall** | 70 (16.63%) |

**Table S2.** Patterns of recurrence

| **Characteristics** | **Univariate** | | | **Multivariate** | | |
| --- | --- | --- | --- | --- | --- | --- |
|  | **Exp(B)** | **95%CI** | **p-value** | **Exp(B)** | **95%CI** | **p-value** |
| **Sex** | 1.495 | 0.878-2.545 | 0.139 |  | | |
| **Age** | 1.310 | 0.768-2.234 | 0.321 |  |  |  |
| **Nodal stage** | 1.169 | 1.040-1.314 | 0.009* | 1.178 | 0.925-1.500 | 0.184 |
| **Tumor stage** | 1.353 | 1.095-1.672 | 0.005* | 1.046 | 0.907-1.206 | 0.536 |
| **Grading** | 2.076 | 1.297-3.322 | 0.002* | 1.692 | 1.021-2.805 | 0.041* |
| **Perineural invasion** | 1.295 | 0.682-2.462 | 0.429 |  | | |
| **Vascular invasion** | 1.193 | 0.165-8.636 | 0.861 |  |  |  |
| **Lymphatic invasion** | 3.751 | 1.591-8.018 | 0.002* | 2.282 | 0.914-5.699 | 0.077 |
| **Resection margin** | 3.078 | 0.747-6.675 | 0.012* | 1.665 | 0.226-3.267 | 0.017* |
| **Localization** | 1.002 | 0.854-1.175 | 0.980 |  | | |

**Table S3.** Uni- and multivariate analyses of local recurrence-free survival in patients with oral squamous cell carcinoma

**Abbreviation:** CI: confidence interval

| **Characteristics** | **Univariate** | | | **Multivariate** | | |
| --- | --- | --- | --- | --- | --- | --- |
|  | **Exp(B)** | **95%CI** | **p-value** | **Exp(B)** | **95%CI** | **p-value** |
| **Sex** | 1.181 | 0.727-1.921 | 0.472 |  | | |
| **Age** | 1.225 | 0.757-1.981 | 0.380 |  |  |  |
| **Nodal stage** | 1.242 | 1.128-1.369 | <0.001* | 1.078 | 0.954-1.218 | 0.231 |
| **Tumor stage** | 1.537 | 1.270-1.860 | <0.001* | 1.339 | 1.079-1.663 | 0.008* |
| **Grading** | 2.381 | 1.550-3.655 | <0.001* | 1.778 | 1.115-2.834 | 0.016* |
| **Perineural invasion** | 1.721 | 1.013-2.924 | 0.044* | 0.777 | 0.431-1.401 | 0.401 |
| **Vascular invasion** | 3.877 | 1.409-10.649 | 0.009* | 1.688 | 0.554-5.137 | 0.357 |
| **Lymphatic invasion** | 5.060 | 2.672-9.575 | <0.001* | 2.668 | 1.211-5.880 | 0.015* |
| **Resection margin** | 2.366 | 0.577-9.690 | 0.228 |  | | |
| **Localization** | 0.997 | 0.865-1.149 | 0.959 |  |  |  |

**Table S4.** Uni- and multivariate analyses of progression-free survival in patients with oral squamous cell carcinoma

**Abbreviation:** CI: confidence interval

**Table S5.** Uni- and multivariate analyses of overall survival in patients with oral squamous cell carcinoma

| **Characteristics** | **Univariate** | | | **Multivariate** | | |
| --- | --- | --- | --- | --- | --- | --- |
|  | **Exp(B)** | **95%CI** | **p-value** | **Exp(B)** | **95%CI** | **p-value** |
| **Sex** | 0.965 | 0.665-1.397 | 0.841 |  | | |
| **Age** | 2.362 | 1.642-3.398 | <0.001* | 2.783 | 1.901-4.074 | <0.001* |
| **Nodal stage** | 1.194 | 1.111-1.282 | <0.001* | 1.075 | 0.980-1.179 | 0.125 |
| **Tumor stage** | 1.295 | 1.126-1.491 | <0.001* | 1.143 | 0.969-1.349 | 0.112 |
| **Grading** | 1.516 | 1.125-2.032 | 0.006* | 1.193 | 0.865-1.645 | 0.281 |
| **Perineural invasion** | 1.597 | 1.073-2.370 | 0.020* | 1.296 | 0.834-2.015 | 0.249 |
| **Vascular invasion** | 2.471 | 1.151-5.316 | 0.020* | 1.434 | 0.619-3.322 | 0.400 |
| **Lymphatic invasion** | 3.454 | 2.152-5.556 | <0.001* | 2.022 | 1.102-3.711 | 0.23 |
| **Resection margin** | 2.962 | 1.202-7.288 | 0.018* | 0.988 | 0.301-3.238 | 0.984 |
| **Localization** | 0.995 | 0.900-1.100 | 0.915 |  | | |

**Abbreviation:** CI: confidence interval
